# Genetically Predicted Gut Microbial Taxa and Inflammatory Proteins Associated With Atrial Fibrillation: A Bidirectional Mendelian Randomization and Colocalization Study

**DOI:** 10.1002/joa3.70454

**Published:** 2026-09-25

**Authors:** Chengcheng Yi, Wenyuan Zheng, Jing Zhao, Weiting Cai, Junqian Wang, Li Song, Ming Bai, Zheng Zhang

**Affiliations:** ^1^ The First Clinical Medical College, Lanzhou University Lanzhou China; ^2^ Heart Center The First Hospital of Lanzhou University Lanzhou China; ^3^ Key Laboratory for Cardiovascular Diseases of Gansu Province The First Hospital of Lanzhou University Lanzhou China

**Keywords:** atrial fibrillation, gastrointestinal microbiome, genetic pleiotropy, inflammation, mendelian randomization analysis

## Abstract

**Background:**

Observational studies have linked the gut microbiota and inflammatory proteins to atrial fibrillation (AF), but confounding, reverse causation, and instrument validity remain concerns.

**Methods:**

We evaluated 473 microbial traits and 91 inflammatory proteins in relation to AF using two GWAS datasets. Sensitivity analyses included instruments selected at *p* < 5 × 10^−8^, MR‐RAPS, *cis*/*trans*‐stratified protein MR, reverse MR, and FGF‐5 colocalization. Participants were predominantly of European ancestry.

**Results:**

Under conventional IVW analysis, six associations were FDR‐significant in the discovery analysis and remained FDR‐significant when selectively evaluated in the second AF GWAS, with FDR correction applied across the six prioritized tests: Leptospirae (OR 0.605, 95% CI 0.457–0.800), Leptospirales (0.499, 0.371–0.673), leukemia inhibitory factor receptor (LIF‐R; 0.905, 0.869–0.943), TWEAK (0.891, 0.856–0.927), FGF‐5 (1.077, 1.051–1.103), and interleukin‐6 (0.886, 0.834–0.942). At *p* < 5 × 10^−8^, neither microbial trait had a harmonized instrument, whereas the protein estimates retained their directions. MR‐RAPS supported LIF‐R in both datasets, but support for the other signals varied. The prioritized proteins also differed in *cis*/*trans* genetic architecture. The FGF‐5 *cis*‐only estimates were based on two harmonized instruments and were therefore preliminary, while *cis*‐region colocalization provided little evidence of a shared variant (PP.H4 = 0.0045).

**Conclusions:**

The conventional IVW associations varied in robustness across alternative analyses. These findings prioritize hypotheses, not established causal pathways or clinical targets. Generalizability beyond predominantly European populations remains uncertain.

## Graphical abstract

A screen prioritized six microbial or inflammatory traits associated with AF by conventional MR and selectively evaluated them in a second AF GWAS. Sensitivity and *cis*/*trans* analyses indicated unequal robustness; the two‐instrument FGF‐5 *cis*‐only estimate remained preliminary; these findings are hypothesis‐generating, not established causal pathways or clinical targets.

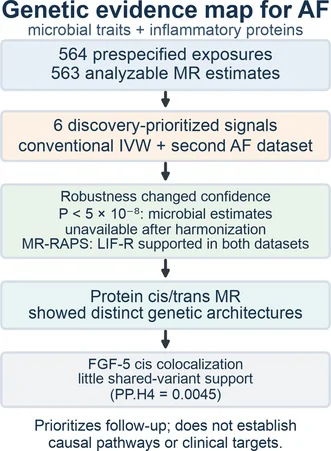

**Abbreviations**
AF: atrial fibrillation
CI: confidence interval
FDR: false discovery rate
FGF‐5: fibroblast growth factor 5
GWAS: genome‐wide association study
IL‐6: interleukin‐6
IVW: inverse‐variance weighted
LD: linkage disequilibrium
Leptospirae: Leptospirae abundance in stool
Leptospirales: Leptospirales abundance in stool
LIF‐R: leukemia inhibitory factor receptor
MR: Mendelian randomization
OR: odds ratio
pQTL: protein quantitative trait locus
SNP: single‐nucleotide polymorphism
TWEAK: tumor necrosis factor ligand superfamily member 12

## 1 Introduction

Atrial fibrillation (AF) is the most common sustained cardiac arrhythmia and a major cause of stroke, heart failure, cognitive decline, mortality, and healthcare expenditure [1, 2, 3]. Large AF genome‐wide association studies (GWASs) have identified susceptibility loci [4, 5], but translating these discoveries into modifiable biological pathways remains challenging. Inflammation, host metabolism, and immune signaling may contribute to atrial remodeling. Observational biomarker studies, however, cannot reliably distinguish causal effects from confounding, medication effects, or disease‐related changes.

The gut microbiome may influence cardiovascular phenotypes through microbial metabolites, immune regulation, endothelial function, and systemic inflammation [6, 7, 8, 9]. Cross‐sectional and prospective studies have linked microbial composition to cardiometabolic risk. Microbiome data are nevertheless susceptible to dietary, environmental, and treatment‐related confounding, including medication‐induced changes in microbial composition [10]. Studies of AF have reported altered microbial metabolic potential and associations between trimethylamine N‐oxide and AF risk [11, 12], although this evidence remains largely observational. Circulating inflammatory proteins offer complementary evidence, as they may reflect host immune responses relevant to AF susceptibility. Observational studies and meta‐analyses have linked inflammatory markers, including IL‐6, to AF occurrence, recurrence, and clinical outcomes [13, 14, 15]. Nevertheless, the direction and specificity of these relationships remain uncertain.

Mendelian randomization (MR) uses exposure‐associated genetic variants as instruments to estimate associations between genetically predicted exposures and disease risk [16, 17]. When the instrumental‐variable assumptions are plausible, MR can reduce confounding and reverse causation. It remains vulnerable to weak instruments, horizontal pleiotropy, population mismatch, and incomplete biological annotation [16, 18, 19, 20]. Multi‐trait analyses using public data therefore require transparent instrument construction, multiplicity control, reverse analyses, evaluation in a separate outcome dataset, and reporting consistent with STROBE‐MR [21, 22].

We reconstructed instruments for 473 gut microbial traits and 91 inflammatory proteins to investigate their genetic associations with AF. After a complete forward screen, we evaluated discovery‐prioritized associations in a second AF outcome dataset. Reverse MR covered the same set of traits. We also assessed instrument strength, harmonization exclusions, MR‐PRESSO, Steiger directionality, stricter instrument thresholds, MR‐RAPS, and *cis*/*trans*‐stratified protein MR. We performed focused regional colocalization for FGF‐5. The study was designed to prioritize testable associations for mechanistic AF research, not to establish a unified microbiome–protein–AF pathway or identify clinical intervention targets.

## 2 Methods

### 2.1 Study Design

**FIGURE 1:**
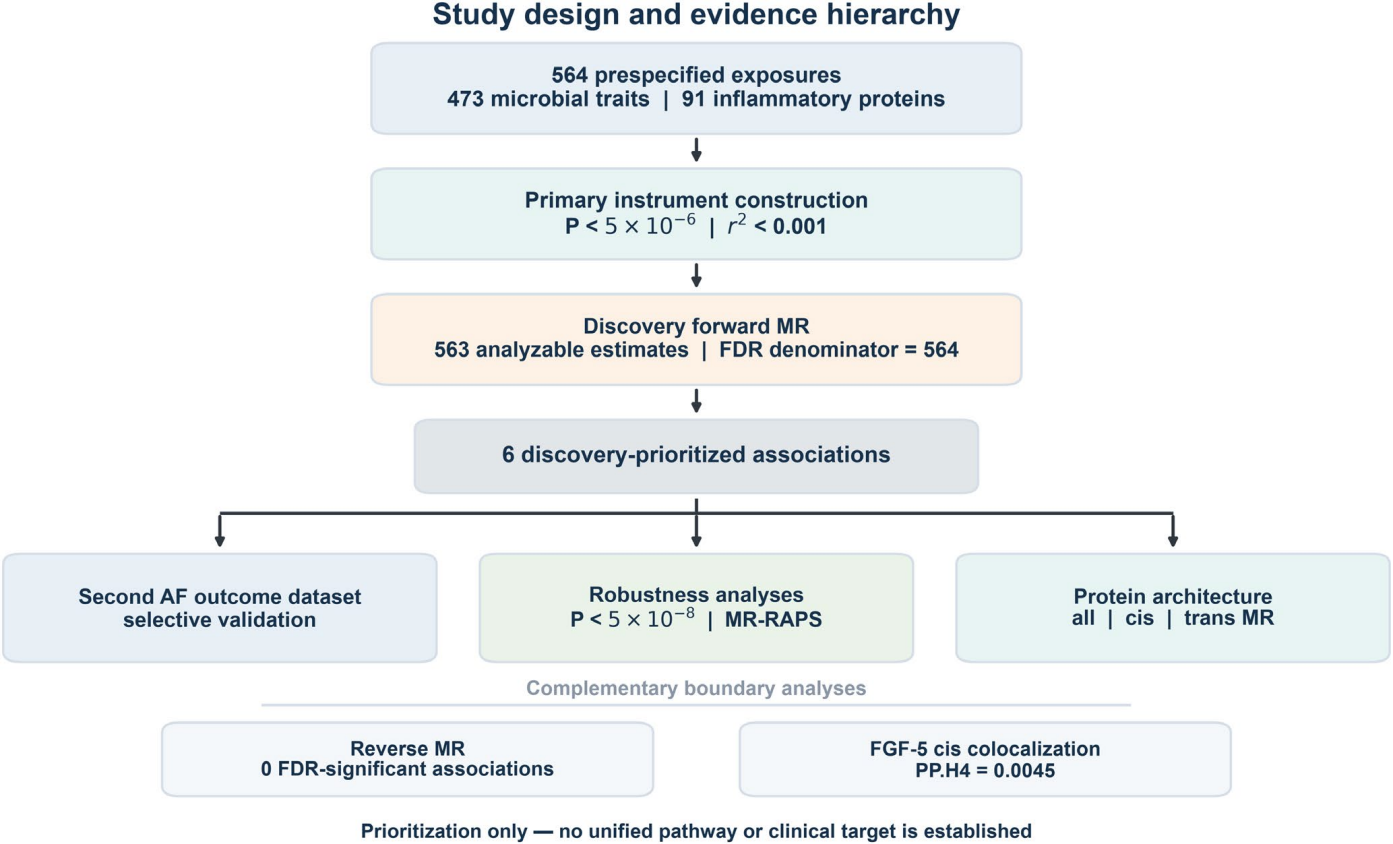
Study design and evidence hierarchy. The workflow shows instrument selection, discovery forward MR, selective validation of discovery‐prioritized associations in a second AF GWAS, reverse MR, strict‐threshold and MR‐RAPS sensitivity analyses, *cis*/*trans*‐stratified protein MR, and focused FGF‐5 colocalization. This workflow prioritizes associations and does not establish a microbiome–protein–AF pathway.

This genetic epidemiology study used summary data to combine two‐sample and bidirectional MR, evaluation in a second AF outcome dataset, and focused colocalization. In the forward analysis, genetically predicted gut microbial taxa or inflammatory protein levels were the exposures and AF was the outcome. In reverse MR, genetically predicted AF liability was the exposure and each microbial or inflammatory trait was an outcome. Figure 1 summarizes the evidence hierarchy, from discovery screening and second‐dataset evaluation to assessment of the limits of mechanistic inference. Interpretation of the main MR estimates depends on three core instrumental‐variable assumptions: instrument relevance, independence from exposure–outcome confounders, and no effect on AF outside the exposure pathway.

### 2.2 Data Sources

Gut microbial GWAS summary statistics covered 473 traits from the FINRISK‐based microbiome GWAS reported by Qin et al. [23]. Related host‐genetic microbiome resources illustrate both the potential and the heterogeneity of microbiome loci [23, 24]. Inflammatory protein GWAS summary statistics covered 91 circulating proteins measured with the Olink inflammation panel, as reported by Zhao et al. [25]. Larger plasma‐proteome pQTL studies provide context for *cis*/*trans* architecture and disease integration [26, 27]. The discovery AF dataset, GCST006414 from Nielsen et al., included 60 620 cases and 970 216 controls of European ancestry [4]. The second AF outcome dataset, GCST006061 from Roselli et al., included 55 114 cases and 482 295 controls of European ancestry [5]. Participant overlap between the AF GWASs could not be quantified from summary‐level metadata. We therefore treated GCST006061 as a separate outcome dataset without assuming full independence. Potential overlap was considered when interpreting reproducibility [28]. All analyses used publicly available summary statistics; no individual‐level data were accessed.

### 2.3 Instrument Selection

Forward instruments were selected from the reconstructed microbiome and inflammatory‐protein summary data at *p* < 5 × 10^−6^. We used this threshold because these GWASs often identify few genome‐wide significant variants for individual traits [23, 25]. Applying a common threshold to both exposure classes preserved broad screening coverage. We used stringent LD clumping, instrument‐strength reporting, FDR correction, and evaluation in a second AF GWAS alongside this threshold. For the six discovery‐prioritized associations, we assessed robustness by repeating MR estimation with instruments restricted to *p* < 5 × 10^−8^, using IVW for multiple instruments and the Wald ratio for a single instrument. No estimate was generated when no variant remained after outcome matching and harmonization. Variants lacking SNP identifiers, alleles, beta estimates, standard errors, or *p* values were excluded before clumping, where applicable. LD clumping used PLINK and the 1000 Genomes European reference panel [29], with *r* ^2^ < 0.001 and a 10 000‐kb window. Variants with a reference‐panel minor allele frequency below 0.01 were removed. Reconstruction yielded 5331 independent microbiome instruments from 51 132 candidate variants and 1820 protein instruments from 86 366 candidate variants. Instrument counts and strength metrics are reported in File S1. These safeguards cannot eliminate weak‐instrument bias or winner's curse, particularly for microbiome traits selected at the suggestive threshold.

### 2.4 Discovery Forward Mendelian Randomization

For each exposure, instrument SNPs were matched to the discovery AF GWAS. Alleles were harmonized so that the AF effect corresponded to the exposure‐increasing allele. We excluded non‐biallelic variants or indels, palindromic A/T or C/G variants, variants absent from the outcome, variants lacking beta or standard‐error values, and allele‐mismatched variants. The forward harmonization audit assessed 7151 SNP–trait instrument rows and retained 5941 harmonized rows. Exclusions comprised 89 variants absent from the AF outcome, 1071 palindromic variants, 49 non‐SNPs or indels, and 1 allele‐mismatched variant. The inverse‐variance weighted (IVW) estimator was the primary method. Weighted median and MR‐Egger estimates were calculated when instrument counts were sufficient [18, 19]. Cochran's *Q* summarized heterogeneity. MR‐Egger intercepts screened for directional pleiotropy. With modest instrument counts, however, limited power meant that a null intercept could not establish the absence of pleiotropy. Related taxa were not treated as biologically independent because taxonomic hierarchy and correlated community features may produce partly redundant signals. The forward screen comprised 564 prespecified exposure–outcome comparisons. Of these, 563 yielded analyzable harmonized estimates and one did not. Benjamini–Hochberg FDR correction conservatively retained the prespecified denominator of 564 [21]. Harmonization exclusions are detailed in File S1: Table S6.

### 2.5 Evaluation in a Second AF Outcome Dataset

All forward associations that survived discovery FDR correction were evaluated in GCST006061 using the same exposure instruments and harmonization rules. Validation required a concordant effect direction and nominal *p* < 0.05; FDR values were calculated across the six tests. This analysis selectively evaluated discovery‐prioritized associations in a second AF GWAS; it did not repeat the complete screen of 564 traits. Because cohort overlap could not be excluded from summary‐level metadata, the analysis was not considered formal replication.

### 2.6 Reverse Mendelian Randomization

AF instruments were selected from the discovery GWAS at genome‐wide significance and LD‐clumped at *r* ^2^ < 0.001 within 10 000 kb, yielding 112 independent instruments. These instruments were tested against all 473 microbial traits and 91 inflammatory proteins. Reverse analyses used the same IVW framework and harmonization rules, with FDR correction across all 564 outcomes. Because power to detect effects differed across outcomes, null results indicated only an absence of statistically robust evidence in this dataset. They were not interpreted as proof that reverse causality was absent [20].

### 2.7 Protein *cis*/*trans* pQTL and FGF‐5 Colocalization Analyses

For LIF‐R, TWEAK, FGF‐5, and IL‐6, clumped pQTL instruments were classified as *cis* if they lay within ±1 Mb of the GRCh37 coordinates of the encoding gene; all other instruments were classified as *trans*. All‐instrument, *cis*‐only, and *trans*‐only IVW estimates were calculated separately in the discovery and second AF datasets when harmonized instruments were available. Subsets containing one variant were analyzed using the Wald ratio and could not support pleiotropy or heterogeneity testing. We also performed regional colocalization for FGF‐5 because matched regional exposure and AF summary data were available. The analysis covered ±1 Mb around the lead FGF5 *cis* pQTL (GRCh37 chromosome 4: 80 164 723–82 164 723; center, 81 164 723). After excluding palindromic variants and indels and harmonizing effect alleles, 2213 SNPs remained. A deposited Python implementation of Wakefield approximate Bayes factors used prior association probabilities *p*_1_ = 1 × 10^−4^, *p*_2_ = 1 × 10^−4^, and *p*_12_ = 1 × 10^−5^. Prior variances were 0.15^2^ for FGF‐5 and 0.20^2^ for AF. Posterior probabilities were estimated for five configurations [30], assuming at most one causal variant for each trait in the region. Regional analysis parameters are reported in File S1: Table S10.

### 2.8 Additional Robustness Analyses

MR‐RAPS was applied to all harmonized instruments for each of the six discovery‐prioritized associations in both AF datasets. Analyses used mr. raps version 0.2 with an overdispersed model, Huber loss, and sandwich standard errors [31]. This analysis assessed sensitivity to weak instruments and idiosyncratic pleiotropy. CAUSE was not implemented because the synchronized repository contained clumped and harmonized instruments, not the aligned genome‐wide exposure and outcome summary data required by the method [32]. Strict‐threshold, MR‐RAPS, and *cis*/*trans* results are reported in File S1.

### 2.9 Statistical Reporting and Software

The deposited Python and R analysis scripts used Python version 3.10, R version 4.4.1, PLINK version 1.9, MRPRESSO version 1.0, and mr.raps version 0.2. For AF outcomes, estimates are reported as log odds ratios and ORs with 95% CIs. Statistical significance in the primary discovery, second‐dataset, and reverse analyses was interpreted after FDR correction. MR‐RAPS sensitivity analyses were interpreted using nominal *p* < 0.05 without FDR correction. MR‐Egger intercepts and Cochran's *Q* summarized directional pleiotropy and heterogeneity, respectively. For the six discovery‐prioritized associations, we generated leave‐one‐out IVW estimates, MR‐PRESSO tests [33], approximate Steiger comparisons [34], strict‐threshold analyses, and MR‐RAPS estimates. Because AF is binary, the Steiger comparisons used variance explained on the observed scale and were interpreted as supportive, not definitive, evidence regarding causal direction. MR‐PRESSO used 1000 simulations, a significance threshold of 0.05, and random seed 20 260 726. Reporting was checked against STROBE‐MR [22]; the official item‐by‐item checklist is provided in File S2. The repository file inventory is provided in File S1: Table S12. The study protocol was not preregistered.

## 3 Results

### 3.1 Instrument Reconstruction and Analytic Coverage

Instrument reconstruction yielded 5331 independent instruments for 473 microbial traits and 1820 instruments for 91 inflammatory proteins. The forward screen comprised 564 prespecified comparisons. Of these, 563 had sufficient harmonized instruments for analysis; one did not. The prespecified denominator of 564 was retained for FDR correction. For every analyzed forward trait, the minimum *F* statistic was at least 10. Among the six FDR‐significant associations, mean *F* statistics ranged from 22.0 for Leptospirae to 105.5 for FGF‐5. The minimum single‐variant *F* statistic exceeded 20 for every prioritized signal. Reverse MR used 112 AF instruments and covered all 564 microbial and inflammatory‐protein outcomes. Complete forward estimates and instrument‐strength metrics are reported in File S1: Tables S3 and S5.

### 3.2 Six Forward Associations Survived FDR Correction

Six genetically predicted exposures were associated with AF after FDR correction in the discovery analysis (Table 1). Leptospirae and Leptospirales were inversely associated with AF risk. Four inflammatory proteins also survived correction: leukemia inhibitory factor receptor (LIF‐R), tumor necrosis factor ligand superfamily member 12 (TWEAK), FGF‐5, and interleukin‐6 (IL‐6). FGF‐5 was positively associated with AF risk, whereas LIF‐R, TWEAK, and IL‐6 were inversely associated. Weighted median and MR‐Egger slope estimates were directionally concordant with the IVW estimates for all six signals. MR‐Egger intercept *p* values ranged from 0.097 for FGF‐5 to 0.807 for IL‐6. These tests provided no strong evidence of directional pleiotropy, but they could not exclude balanced pleiotropy or pleiotropy that the tests had limited power to detect. Approximate observed‐scale Steiger comparisons were consistent with the exposure‐to‐AF direction for all six signals, but, because AF is binary, they were considered supportive rather than definitive evidence of causal direction. MR‐PRESSO global tests indicated horizontal pleiotropy for Leptospirae, Leptospirales, and TWEAK. The outlier tests identified two SNPs for each microbial trait and one for TWEAK (File S1: Table S14). Leave‐one‐out directional consistency is reported in File S1. Directional consistency does not mean that every leave‐one‐out estimate remained statistically significant. These results prioritize associations but do not establish direct biological mechanisms. Discovery estimates, leave‐one‐out analyses and approximate Steiger comparisons are reported in File S1: Tables S1, S7, and S13.

**TABLE 1:** Discovery forward Mendelian randomization associations surviving FDR correction across 564 prespecified comparisons.

Exposure	Family	SNPs	OR (95% CI)	*p*	FDR
Leptospirae	Microbiome	13	0.605 (0.457–0.800)	4.27e‐04	0.040
Leptospirales	Microbiome	11	0.499 (0.371–0.673)	4.85e‐06	6.83e‐04
LIF‐R	Protein	16	0.905 (0.869–0.943)	1.86e‐06	3.49e‐04
TWEAK	Protein	24	0.891 (0.856–0.927)	9.54e‐09	2.69e‐06
FGF‐5	Protein	20	1.077 (1.051–1.103)	2.34e‐09	1.32e‐06
IL‐6	Protein	8	0.886 (0.834–0.942)	1.08e‐04	0.012

*Note:* Full exposure names are provided in the Abbreviations and File S1. ORs represent AF risk per genetically predicted increment in each exposure. The Benjamini–Hochberg denominator was the prespecified family of 564 forward comparisons, of which 563 yielded analyzable estimates.

### 3.3 Sensitivity to Instrument Threshold and MR‐RAPS

At *p* < 5 × 10^−8^, Leptospirae and Leptospirales each retained one exposure‐associated variant, but neither was available after AF outcome matching and harmonization. Strict‐threshold MR estimates were therefore unavailable for either microbial signal. LIF‐R retained three harmonized instruments in each AF dataset (discovery OR = 0.887, 95% CI 0.841–0.935; second AF GWAS OR = 0.908, 95% CI 0.859–0.960). TWEAK retained six and five instruments (0.856, 0.812–0.902; and 0.836, 0.789–0.886), respectively. FGF‐5 retained two instruments in each dataset (1.089, 1.060–1.119; and 1.076, 1.045–1.108). IL‐6 retained one instrument, yielding Wald‐ratio estimates of 0.790 (0.730–0.856) and 0.796 (0.731–0.866). The single‐variant IL‐6 analysis could not assess pleiotropy (Figure 3a). Strict‐threshold estimates are reported in File S1: Table S16.

MR‐RAPS support varied across the six associations. LIF‐R remained associated with AF in both datasets (discovery OR = 0.904, 95% CI 0.861–0.949, *p* = 4.30 × 10^−5^; second AF GWAS OR = 0.916, 95% CI 0.871–0.963, *p* = 6.13 × 10^−4^). MR‐RAPS provided nominal support for TWEAK and FGF‐5 in discovery but not in the second AF GWAS. Leptospirae, Leptospirales, and IL‐6 were not supported in either dataset. Thus, the six conventional IVW associations differed in robustness under weak‐instrument and pleiotropy‐robust modeling (Figure 3b and File S1). Full MR‐RAPS estimates are reported in File S1: Table S17.

### 3.4 Validation of Discovery‐Prioritized Associations in a Second AF GWAS

**FIGURE 2:**
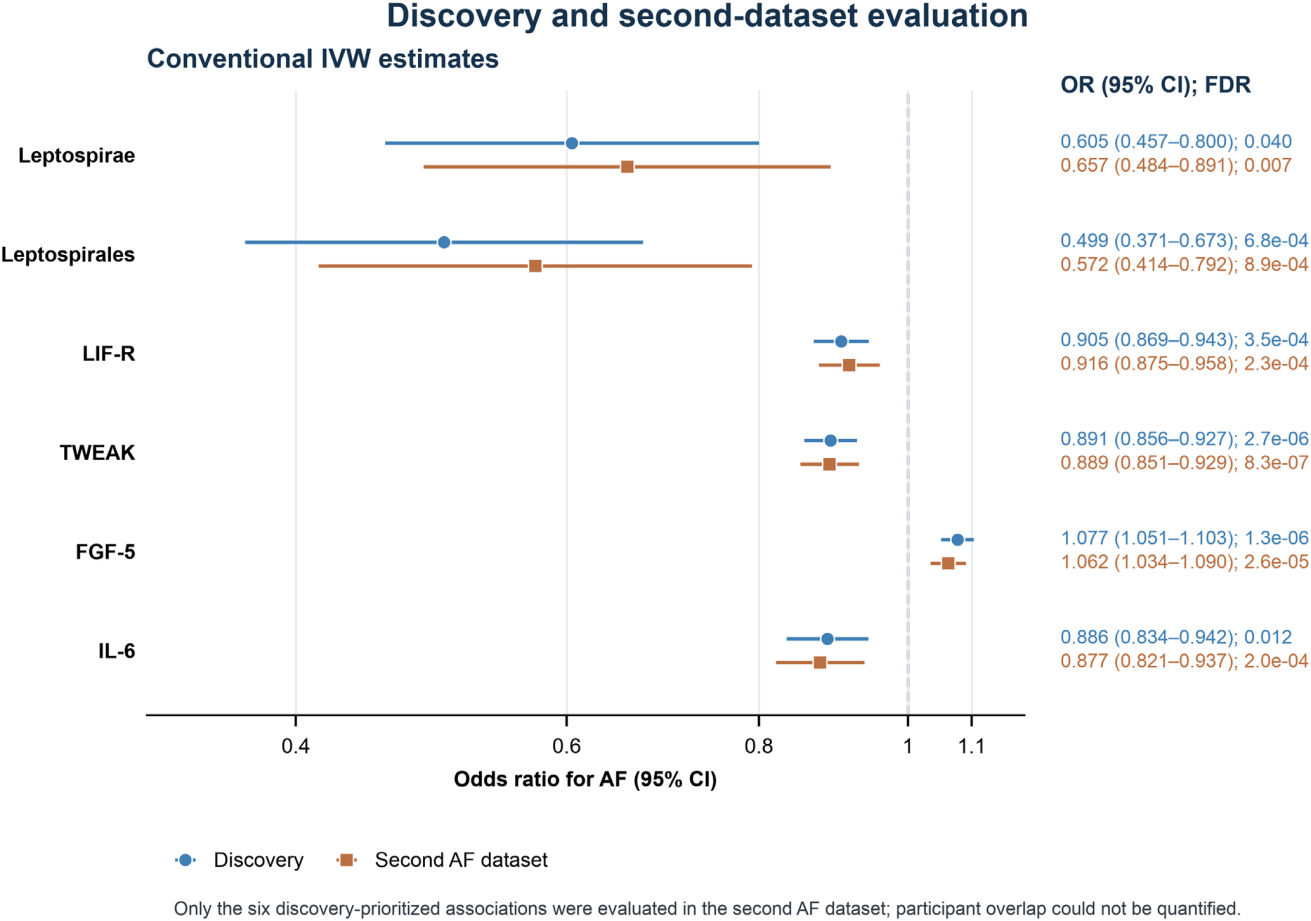
Discovery and second‐dataset evaluation of forward MR associations. The forest plot shows conventional IVW estimates and 95% CIs for the six discovery‐prioritized associations in the discovery AF GWAS (circles) and their selective evaluation in GCST006061 (squares). FDR values were calculated across 564 prespecified discovery comparisons and six second‐dataset tests, respectively. The analysis is not described as formal replication because cohort overlap could not be quantified and only discovery‐prioritized associations were evaluated.

Under conventional IVW analysis, all six discovery‐prioritized associations were directionally concordant and remained FDR‐significant when evaluated in the second AF GWAS, with FDR correction applied across these six selective tests (Table 2 and Figure 2). This selective validation reduced reliance on the discovery AF dataset but did not constitute formal replication of the complete 564‐trait screen. Only discovery‐prioritized associations were tested, and cohort overlap could not be quantified. MR‐RAPS results were less consistent across the two datasets. The six selective second‐dataset estimates are reported in File S1: Table S2.

**TABLE 2:** Evaluation in the second AF outcome dataset (GCST006061).

Exposure	SNPs	Second‐dataset OR (95% CI)	*p*	FDR	Same direction
Leptospirae	13	0.657 (0.484–0.891)	0.007	0.007	Yes
Leptospirales	11	0.572 (0.414–0.792)	7.45e‐04	8.94e‐04	Yes
LIF‐R	13	0.916 (0.875–0.958)	1.52e‐04	2.27e‐04	Yes
TWEAK	20	0.889 (0.851–0.929)	1.38e‐07	8.30e‐07	Yes
FGF‐5	19	1.062 (1.034–1.090)	8.59e‐06	2.58e‐05	Yes
IL‐6	8	0.877 (0.821–0.937)	9.81e‐05	1.96e‐04	Yes

*Note:* GCST006061 was used for selective validation of the six discovery‐prioritized associations. FDR correction was applied across these six tests. Because participant overlap could not be quantified and the complete 564‐trait screen was not repeated, this analysis is not described as formal replication.

### 3.5 Reverse MR Did Not Support Robust AF‐To‐Trait Effects

Reverse MR used 112 independent AF instruments and covered 564 outcomes. No association survived FDR correction across the 473 microbial traits and 91 inflammatory proteins. The analyses generated 54 704 harmonized SNP–outcome rows. The smallest nominal *p* values were observed for Provencibacterium abundance, FGF‐5 levels, and UBA2922 sp900313925 abundance. Each association had BH/FDR = 0.623, so none survived multiplicity correction. The reverse screen identified no statistically robust AF‐to‐trait association. However, limited power, which varied by outcome, means that these null findings cannot exclude reverse causality. Complete reverse MR estimates are reported in File S1: Table S4.

### 3.6 Protein *cis*/*trans* Architecture and FGF‐5 Colocalization

**FIGURE 3:**
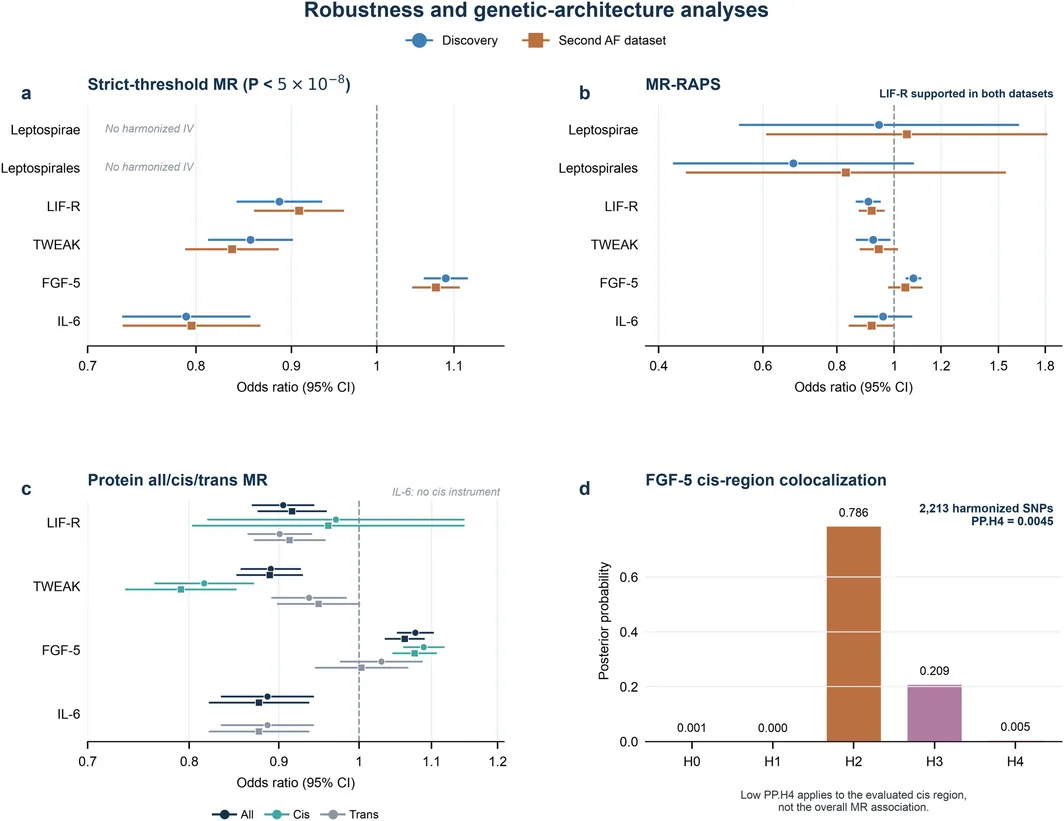
Robustness and genetic‐architecture analyses. (a) Strict‐threshold MR estimates based on instruments selected at *p* < 5 × 10^−8^, using IVW for multiple instruments and the Wald ratio for single‐variant analyses. Leptospirae and Leptospirales had no harmonized strict‐threshold instrument, so estimates were unavailable. (b) MR‐RAPS estimates for the six discovery‐prioritized associations in the discovery and second AF datasets. (c) All‐instrument, *cis*‐only, and *trans*‐only MR estimates for LIF‐R, TWEAK, FGF‐5, and IL‐6. Circles denote discovery estimates and squares denote second‐AF‐dataset estimates; IL‐6 had no *cis* instrument. *cis*‐only estimates based on one or two instruments are preliminary and cannot assess pleiotropy. (d) FGF‐5 *cis*‐region colocalization posterior probabilities based on 2213 harmonized SNPs. The low PP.H4 applies only to the evaluated *cis* region and neither confirms nor excludes the overall FGF‐5 MR association. Points and horizontal lines in Panels a–c indicate ORs and 95% CIs.

The four prioritized proteins differed in *cis*/*trans* architecture. LIF‐R included one harmonized *cis* instrument and 15 *trans* instruments in discovery. Its *cis*‐only estimate was imprecise and null, whereas the *trans*‐only estimate resembled the all‐instrument result. TWEAK included two *cis* instruments and 22 *trans* instruments. Its *cis*‐only association was supported in both AF datasets, whereas the *trans*‐only association was weaker and not significant in the second dataset. FGF‐5 had 23 clumped instruments, comprising 3 *cis* and 20 *trans* variants; 2 *cis* and 18 *trans* instruments were harmonized in discovery. Its two‐instrument *cis*‐only estimate was statistically supported in both AF datasets, whereas its *trans*‐only estimate was null in both. However, the *cis*‐only estimate was highly preliminary because two instruments could not meaningfully assess horizontal pleiotropy or heterogeneity. IL‐6 had no *cis* instrument and was therefore entirely *trans*‐driven. These results distinguish protein‐level association signals from evidence consistent with direct *cis* regulation (Figure 3c and File S1). Regional FGF‐5 colocalization included 2213 harmonized SNPs and provided little support for a shared variant at the evaluated *cis* locus (PP.H4 = 0.0045; Figure 3d). The low PP.H4 neither confirms nor excludes the causal basis of the overall FGF‐5 MR association because colocalization and all‐instrument MR assessed different components of its genetic architecture. Instrument classification, FGF‐5 regional data and *cis*/*trans* estimates are provided in File S1: Tables S8, S9, S15, S18 and S19.

## 4 Discussion

The conventional IVW screen identified six microbial or inflammatory traits associated with AF, and selective validation in a second AF GWAS reproduced their effect directions. These signals varied in robustness across additional analyses. At genome‐wide significance, no harmonized instruments remained for the two microbial traits. MR‐RAPS consistently supported only LIF‐R across both AF datasets. *cis*/*trans*‐stratified analyses further distinguished protein associations with *cis*, *trans*, or mixed genetic architectures. The findings therefore prioritize testable associations: they do not establish causal proteins, microorganisms, or a unified biological pathway.

Leptospirae and Leptospirales were inversely associated with AF in the primary IVW analysis, but neither could be estimated at *p* < 5 × 10^−8^ after harmonization. Neither association was supported by MR‐RAPS. Limited heritability, environmental sensitivity, compositional structure, and taxonomic relatedness constrain their causal interpretation [23, 24]. These related traits should not be counted as two independent biological discoveries or taken as evidence of directly protective microorganisms. Instead, they may reflect a correlated host–microbial ecological or community signature that warrants further investigation.

The inflammatory‐protein findings must also be interpreted in light of their genetic architecture. LIF‐R was supported by MR‐RAPS in both datasets, but its all‐instrument association was predominantly *trans*‐driven; the single *cis* estimate was imprecise and null. TWEAK showed consistent *cis*‐only estimates, whereas its *trans*‐only estimate was weaker. IL‐6 had no *cis* instrument and was not supported by MR‐RAPS in either dataset, although the second‐dataset estimate narrowly missed nominal significance (*p* = 0.052). The inverse IL‐6 estimate should not be taken as evidence of clinical protection or extrapolated to pharmacological modulation. Circulating pQTLs may affect assay binding or upstream networks rather than tissue‐specific protein activity [25, 26, 27]. These findings identify protein‐level genetic signals for follow‐up rather than treatment‐effect estimates or confirmed direct protein effects.

FGF‐5 illustrates the distinction between an MR association and mechanistic localization. Most clumped instruments were *trans*, but the *trans*‐only estimate was null. The two‐instrument *cis*‐only estimate was supported in both AF datasets. MR‐RAPS supported FGF‐5 in discovery but not in the second dataset. Colocalization provided little evidence of a shared causal variant at the evaluated FGF5 *cis* locus. This result does not disprove the overall MR association. Conversely, the *cis*‐only estimate does not confirm a *cis*‐mediated causal effect because only two harmonized *cis* instruments were available. FGF‐5 therefore remains a hypothesis‐generating signal requiring further genetic and experimental validation, not a confirmed causal protein target.

We did not perform a formal microbiome–protein–AF mediation analysis. The microbial and protein findings are parallel prioritization signals and do not establish a microbiome → inflammatory protein → AF pathway. Demonstrating such a mechanistic link would require an explicit mediation analysis based on consistently harmonized data and appropriate multiplicity control. The exclusion of superseded mediation outputs is documented in File S1: Table S11.

### 4.1 Implications for Arrhythmia Research

These findings primarily guide hypothesis generation. The results prioritize microbial‐community features and inflammatory‐protein signals for mechanistic studies of AF susceptibility. They do not support microbiome modification, cytokine targeting, or FGF‐5‐directed treatment as clinical interventions. Future studies should incorporate stronger *cis* instruments, independent cohorts and ancestries, tissue‐relevant molecular data, and experimental models.

The study has several strengths. We used a prespecified screening framework, FDR correction across 564 comparisons, selective validation in a second AF GWAS, and a complete reverse screen. We also performed strict‐threshold and MR‐RAPS analyses and classified every prioritized protein instrument as *cis* or *trans*. These analyses revealed differences that were not apparent from conventional IVW, weighted median, MR‐Egger, leave‐one‐out, and MR‐PRESSO summaries alone.

This study has several limitations. Participant overlap between exposure and AF GWASs, and between the two AF datasets, could not be quantified [28]. The second‐dataset analysis was selective, evaluating only the six discovery‐prioritized associations. Most participants were of European ancestry. The microbiome traits had limited heritability, modest sample sizes, and substantial environmental and measurement variability [23, 24]. At *p* < 5 × 10^−8^, neither microbial trait had a harmonized instrument, precluding strict‐threshold estimation. MR‐RAPS did not consistently support five of the six signals across both datasets, indicating sensitivity to model choice and possible weak‐instrument or pleiotropic effects. Protein estimates also depended on *cis*/*trans* architecture. LIF‐R and IL‐6 were *trans*‐driven, and several *cis*‐only estimates relied on one or two variants. CAUSE was not implemented because aligned genome‐wide exposure and outcome data were unavailable in the synchronized analysis package [32]. Reverse MR remained limited by outcome‐specific power. FGF‐5 *cis*‐region colocalization could neither confirm nor exclude the causal basis of the overall MR association. These limitations warrant cautious, hypothesis‐generating interpretation.

## 5 Conclusions

Conventional IVW analyses identified six discovery‐prioritized associations and reproduced their effect directions in a second AF GWAS; FDR correction in the second dataset was limited to these six selective tests rather than a repeated 564‐trait screen. However, strict‐threshold, MR‐RAPS, and *cis*/*trans*‐stratified analyses revealed unequal robustness and distinct genetic architectures. Only LIF‐R retained MR‐RAPS support in both AF datasets, and its signal was predominantly *trans*‐driven. The microbial findings lacked harmonized genome‐wide significant instruments. The two‐instrument FGF‐5 *cis*‐only estimate was preliminary and could not meaningfully assess pleiotropy or heterogeneity. *cis*‐region colocalization neither confirmed nor excluded the overall association. These findings provide hypothesis‐generating priorities for AF research rather than established causal pathways, direct protein effects, or clinical targets.

## Funding

This work was supported by the First Hospital of Lanzhou University (grant number ldyyyn2020‐51). The funder had no role in study design, data analysis, manuscript preparation, or the decision to submit the manuscript.

## Ethics Statement

This study used only publicly available, de‐identified GWAS summary statistics. Ethical approval and informed consent were obtained for the original studies. No new individual‐level participant data were collected or accessed, so no additional institutional ethical approval was required.

## Consent

Participants provided informed consent in the original GWAS studies. This study involved no new participant contact or access to individual‐level data.

## Conflicts of Interest

The authors declare no conflicts of interest.

## Supporting information


**File S1:** Table S1: Six forward MR associations significant after BH/FDR correction.
**Table S2:** Evaluation in a second AF outcome dataset using Roselli et al. summary statistics; complete participant independence could not be established.
**Table S3:** Full forward screen of microbiome and protein traits, including SNP number, *R* ^2^, *F* statistics, IVW, weighted median, MR‐Egger, intercept and Cochran *Q*.
**Table S4:** Full AF‐to‐trait reverse MR, including harmonization exclusion counts.
**Table S5:** Trait‐level instrument strength and descriptive power metrics.
**Table S6:** Forward instrument matching and harmonization exclusion counts reconstructed from local inputs.
**Table S7:** Leave‐one‐out IVW sensitivity analysis for the six FDR‐significant discovery associations.
**Table S8:** FGF‐5 clumped instruments classified as *cis*/*trans*.
**Table S9:** Regional FGF‐5 pQTL‐AF harmonized SNPs for coloc.
**Table S10:** Colocalization priors, window, SNP count, and interpretation boundary.
**Table S11:** Superseded step1‐6 mediation files excluded from rebuilt‐only submission package.
**Table S12:** Repository manifest and submission‐facing file status.
**Table S13:** Approximate observed‐scale Steiger directionality comparison for the six FDR‐significant discovery signals.
**Table S14:** MR‐PRESSO global/outlier/distortion results for the six FDR‐significant discovery signals.
**Table S15:** FGF‐5 *cis*‐only IVW estimate and comparison with all harmonized instruments.
**Table S16:** Strict‐threshold MR (*p* < 5 × 10^−8^): IVW for multiple instruments, Wald ratio for one; zero harmonized instruments are reported explicitly.
**Table S17:** MR‐RAPS in both AF datasets (overdispersion, Huber loss, sandwich SE); nominal *p* < 0.05, without FDR correction.
**Table S18:** Prioritized inflammatory‐protein instruments classified as *cis* (within ±1 Mb of the encoding gene, GRCh37) or *trans*.
**Table S19:** All/*cis*/*trans* MR in both AF datasets: IVW for multiple instruments and Wald ratio for a single instrument.

**File S2:** STROBE MR official item by item checklist.

## Data Availability

All summary statistics are publicly available from the GWAS Catalog or repositories cited by the original studies. The discovery and second AF outcome datasets were GCST006414 and GCST006061, respectively. Reconstructed results, analysis scripts, and figure files are available through Zenodo (https://doi.org/10.5281/zenodo.22308382).

## References

[1] J. A. Joglar , M. K. Chung , A. L. Armbruster , et al., “2023 ACC/AHA/ACCP/HRS Guideline for the Diagnosis and Management of Atrial Fibrillation: A Report of the American College of Cardiology/American Heart Association Joint Committee on Clinical Practice Guidelines,” Circulation 149 (2024): e1–e156, 10.1161/CIR.0000000000001193. PMC11095842 38033089

[2] S. S. Chugh , R. Havmoeller , K. Narayanan , et al., “Worldwide Epidemiology of Atrial Fibrillation: A Global Burden of Disease 2010 Study,” Circulation 129 (2014): 837–847, 10.1161/CIRCULATIONAHA.113.005119. PMC4151302 24345399

[3] R. B. Schnabel , X. Yin , P. Gona , et al., “50 Year Trends in Atrial Fibrillation Prevalence, Incidence, Risk Factors, and Mortality in the Framingham Heart Study: A Cohort Study,” Lancet 386 (2015): 154–162, 10.1016/S0140-6736(14)61774-8. PMC4553037 25960110

[4] J. B. Nielsen , R. B. Thorolfsdottir , L. G. Fritsche , et al., “Biobank‐Driven Genomic Discovery Yields New Insight Into Atrial Fibrillation Biology,” Nature Genetics 50 (2018): 1234–1239, 10.1038/s41588-018-0171-3. PMC6530775 30061737

[5] C. Roselli , M. D. Chaffin , L. C. Weng , et al., “Multi‐Ethnic Genome‐Wide Association Study for Atrial Fibrillation,” Nature Genetics 50 (2018): 1225–1233, 10.1038/s41588-018-0133-9. PMC6136836 29892015

[6] W. H. W. Tang , D. Y. Li , and S. L. Hazen , “Dietary Metabolism, the Gut Microbiome, and Heart Failure,” Nature Reviews. Cardiology 16 (2019): 137–154, 10.1038/s41569-018-0108-7. PMC6377322 30410105

[7] R. M. Chakaroun , L. M. Olsson , and F. Backhed , “The Potential of Tailoring the Gut Microbiome to Prevent and Treat Cardiometabolic Disease,” Nature Reviews. Cardiology 20 (2023): 217–235, 10.1038/s41569-022-00771-0. 36241728

[8] N. Kazemian , M. Mahmoudi , F. Halperin , J. C. Wu , and S. Pakpour , “Gut Microbiota and Cardiovascular Disease: Opportunities and Challenges,” Microbiome 8 (2020): 36, 10.1186/s40168-020-00821-0. PMC7071638 32169105

[9] Z. Jie , H. Xia , S. L. Zhong , et al., “The Gut Microbiome in Atherosclerotic Cardiovascular Disease,” Nature Communications 8 (2017): 845, 10.1038/s41467-017-00900-1. PMC5635030 29018189

[10] A. Vich Vila , V. Collij , S. Sanna , et al., “Impact of Commonly Used Drugs on the Composition and Metabolic Function of the Gut Microbiota,” Nature Communications 11 (2020): 362, 10.1038/s41467-019-14177-z. PMC6969170 31953381

[11] K. Zuo , X. Liu , P. Wang , et al., “Metagenomic Data‐Mining Reveals Enrichment of Trimethylamine‐N‐Oxide Synthesis in Gut Microbiome in Atrial Fibrillation Patients,” BMC Genomics 21 (2020): 526, 10.1186/s12864-020-06944-w. PMC7391570 32731896

[12] W. T. Yang , R. Yang , Q. Zhao , X. D. Li , and Y. T. Wang , “A Systematic Review and Meta‐Analysis of the Gut Microbiota‐Dependent Metabolite Trimethylamine N‐Oxide With the Incidence of Atrial Fibrillation,” Annals of Palliative Medicine 10 (2021): 11512–11523, 10.21037/apm-21-2763. 34872276

[13] N. Nso , K. R. Bookani , M. Metzl , and F. Radparvar , “Role of Inflammation in Atrial Fibrillation: A Comprehensive Review of Current Knowledge,” Journal of Arrhythmia 37 (2021): 1–10, 10.1002/joa3.12473. PMC7896450 33664879

[14] N. Wu , B. Xu , Y. Xiang , et al., “Association of Inflammatory Factors With Occurrence and Recurrence of Atrial Fibrillation: A Meta‐Analysis,” International Journal of Cardiology 169 (2013): 62–72, 10.1016/j.ijcard.2013.08.078. 24095158

[15] P. Zhou , M. Waresi , Y. Zhao , et al., “Increased Serum Interleukin‐6 Level as a Predictive Biomarker for Atrial Fibrillation: A Systematic Review and Meta‐Analysis,” Revista Portuguesa de Cardiologia 39 (2020): 723–728, 10.1016/j.repc.2020.07.009. 33234354

[16] G. Davey Smith and S. Ebrahim , “'Mendelian Randomization': Can Genetic Epidemiology Contribute to Understanding Environmental Determinants of Disease?,” International Journal of Epidemiology 32 (2003): 1–22, 10.1093/ije/dyg070. 12689998

[17] G. Hemani , J. Zheng , B. Elsworth , et al., “The MR‐Base Platform Supports Systematic Causal Inference Across the Human Phenome,” eLife 7 (2018): e34408, 10.7554/eLife.34408. PMC5976434 29846171

[18] J. Bowden , G. Davey Smith , and S. Burgess , “Mendelian Randomization With Invalid Instruments: Effect Estimation and Bias Detection Through Egger Regression,” International Journal of Epidemiology 44 (2015): 512–525, 10.1093/ije/dyv080. PMC4469799 26050253

[19] J. Bowden , G. Davey Smith , P. C. Haycock , and S. Burgess , “Consistent Estimation in Mendelian Randomization With Some Invalid Instruments Using a Weighted Median Estimator,” Genetic Epidemiology 40 (2016): 304–314, 10.1002/gepi.21965. PMC4849733 27061298

[20] S. Burgess and S. G. Thompson , “Avoiding Bias From Weak Instruments in Mendelian Randomization Studies,” International Journal of Epidemiology 40 (2011): 755–764, 10.1093/ije/dyr036. 21414999

[21] Y. Benjamini and Y. Hochberg , “Controlling the False Discovery Rate: A Practical and Powerful Approach to Multiple Testing,” Journal of the Royal Statistical Society, Series B: Statistical Methodology 57 (1995): 289–300, 10.1111/j.2517-6161.1995.tb02031.x.

[22] V. W. Skrivankova , R. C. Richmond , B. A. R. Woolf , et al., “Strengthening the Reporting of Observational Studies in Epidemiology Using Mendelian Randomization: The STROBE‐MR Statement,” Journal of the American Medical Association 326 (2021): 1614–1621, 10.1001/jama.2021.18236. 34698778

[23] Y. Qin , A. S. Havulinna , Y. Liu , et al., “Combined Effects of Host Genetics and Diet on Human Gut Microbiota and Incident Disease in a Single Population Cohort,” Nature Genetics 54 (2022): 134–142, 10.1038/s41588-021-00991-z. PMC9883041 35115689

[24] A. Kurilshikov , C. Medina‐Gomez , R. Bacigalupe , et al., “Large‐Scale Association Analyses Identify Host Factors Influencing Human Gut Microbiome Composition,” Nature Genetics 53 (2021): 156–165, 10.1038/s41588-020-00763-1. PMC8515199 33462485

[25] J. H. Zhao , D. Stacey , N. Eriksson , et al., “Genetics of Circulating Inflammatory Proteins Identifies Drivers of Immune‐Mediated Disease Risk and Therapeutic Targets,” Nature Immunology 24 (2023): 1540–1551, 10.1038/s41590-023-01588-w. PMC10457199 37563310

[26] B. B. Sun , J. C. Maranville , J. E. Peters , et al., “Genomic Atlas of the Human Plasma Proteome,” Nature 558 (2018): 73–79, 10.1038/s41586-018-0175-2. PMC6697541 29875488

[27] E. Ferkingstad , P. Sulem , B. A. Atlason , et al., “Large‐Scale Integration of the Plasma Proteome With Genetics and Disease,” Nature Genetics 53 (2021): 1712–1721, 10.1038/s41588-021-00978-w. 34857953

[28] S. Burgess , N. M. Davies , and S. G. Thompson , “Bias due to Participant Overlap in Two‐Sample Mendelian Randomization,” Genetic Epidemiology 40 (2016): 597–608, 10.1002/gepi.21998. PMC5082560 27625185

[29] 1000 Genomes Project Consortium , A. Auton , L. D. Brooks , R. M. Durbin , et al., “A Global Reference for Human Genetic Variation,” Nature 526 (2015): 68–74, 10.1038/nature15393. PMC4750478 26432245

[30] C. Giambartolomei , D. Vukcevic , E. E. Schadt , et al., “Bayesian Test for Colocalisation Between Pairs of Genetic Association Studies Using Summary Statistics,” PLoS Genetics 10 (2014): e1004383, 10.1371/journal.pgen.1004383. PMC4022491 24830394

[31] Q. Zhao , J. Wang , G. Hemani , J. Bowden , and D. S. Small , “Statistical Inference in Two‐Sample Summary‐Data Mendelian Randomization Using Robust Adjusted Profile Score,” Annals of Statistics 48 (2020): 1742–1769, 10.1214/19-AOS1866.

[32] J. Morrison , N. Knoblauch , J. H. Marcus , M. Stephens , and X. He , “Mendelian Randomization Accounting for Correlated and Uncorrelated Pleiotropic Effects Using Genome‐Wide Summary Statistics,” Nature Genetics 52 (2020): 740–747, 10.1038/s41588-020-0631-4. PMC7343608 32451458

[33] M. Verbanck , C. Y. Chen , B. Neale , and R. Do , “Detection of Widespread Horizontal Pleiotropy in Causal Relationships Inferred From Mendelian Randomization Between Complex Traits and Diseases,” Nature Genetics 50 (2018): 693–698, 10.1038/s41588-018-0099-7. PMC6083837 29686387

[34] G. Hemani , K. Tilling , and G. Davey Smith , “Orienting the Causal Relationship Between Imprecisely Measured Traits Using GWAS Summary Data,” PLoS Genetics 13 (2017): e1007081, 10.1371/journal.pgen.1007081. PMC5711033 29149188

