# Supplementary material for: Genetically Predicted Gut Microbial Taxa and Inflammatory Proteins Associated With Atrial Fibrillation: A Bidirectional Mendelian Randomization and Colocalization Study
Source: J Arrhythm. 2026 Sep 25;42(5):e70454. doi: 10.1002/joa3.70454 (PMC13613428; doi:10.1002/joa3.70454)
Supplement: Supplementary file 1 [file JOA3-42-e70454-s002.docx]

Data S2 STROBE MR official item by item checklist

| **Item No.** | **Section** | **Checklist item** | Main manuscript location and reporting note |
| --- | --- | --- | --- |
| 1 | **TITLE and ABSTRACT** | Indicate Mendelian randomization (MR) as the study’s design in the title and/or the abstract if that is a main purpose of the study | Title; Abstract Bidirectional MR and colocalization named in the title and Abstract. |
|  | **INTRODUCTION** |  |  |
| 2 | **Background** | Explain the scientific background and rationale for the reported study. What is the exposure? Is a potential causal relationship between exposure and outcome plausible? Justify why MR is a helpful method to address the study question | Introduction Background, AF rationale, microbiome and inflammatory-protein exposures, and MR motivation. |
| 3 | **Objectives** | State specific objectives clearly, including pre-specified causal hypotheses (if any). State that MR is a method that, under specific assumptions, intends to estimate causal effects | Introduction, final paragraph; Methods, Study design Forward, reverse and second-AF-dataset objectives; assumptions qualify interpretation. |
|  | **METHODS** |  |  |
| 4 | **Study design and data sources** | Present key elements of the study design early in the article. Consider including a table listing sources of data for all phases of the study. For each data source contributing to the analysis, describe the following: | Methods, Study design; Figure 1 Two-sample forward and reverse MR, selective second-dataset evaluation, and focused colocalization. |
|  | a) | Setting: Describe the study design and the underlying population, if possible. Describe the setting, locations, and relevant dates, including periods of recruitment, exposure, follow-up, and data collection, when available. | Methods, Data sources FINRISK microbiome, Olink proteins, discovery AF and second AF GWAS sources and ancestry. |
|  | b) | Participants: Give the eligibility criteria, and the sources and methods of selection of participants. Report the sample size, and whether any power or sample size calculations were carried out prior to the main analysis | Methods, Data sources; original GWAS reports [4, 5, 23, 25] AF case and control counts are given; source-cohort recruitment and eligibility are in cited original studies. No new power calculation is reported. |
|  | c) | Describe measurement, quality control and selection of genetic variants | Methods, Instrument selection; Discovery forward MR; Additional file 1, S5–S6 Variant eligibility, LD clumping, European reference panel, harmonization, and strength metrics. |
|  | d) | For each exposure, outcome, and other relevant variables, describe methods of assessment and diagnostic criteria for diseases | Methods, Data sources Microbiome, circulating Olink protein and binary AF phenotypes are defined by the cited GWAS datasets. |
|  | e) | Provide details of ethics committee approval and participant informed consent, if relevant | Declarations, Approval of research protocol; Informed consent Public, de-identified summary data only; original studies obtained ethics approval and consent. |
| 5 | **Assumptions** | Explicitly state the three core IV assumptions for the main analysis (relevance, independence and exclusion restriction) as well assumptions for any additional or sensitivity analysis | Methods, Study design; Instrument selection; Discovery forward MR Relevance, independence and exclusion-restriction assumptions are explicitly stated; F statistics and pleiotropy diagnostics address only aspects of their validity. |
| 6 | **Statistical methods: main analysis** | Describe statistical methods and statistics used | Methods, Discovery forward MR; Statistical reporting and software Primary IVW, uncertainty, heterogeneity, pleiotropy, and FDR methods. |
|  | a) | Describe how quantitative variables were handled in the analyses (i.e., scale, units, model) | Methods, Statistical reporting and software AF effects expressed as log odds ratios and odds ratios with 95% confidence intervals. |
|  | b) | Describe how genetic variants were handled in the analyses and, if applicable, how their weights were selected | Methods, Instrument selection; Discovery forward MR Clumping, harmonization, equal instrument use and IVW inverse-variance weighting. |
|  | c) | Describe the MR estimator (e.g. two-stage least squares, Wald ratio) and related statistics. Detail the included covariates and, in case of two-sample MR, whether the same covariate set was used for adjustment in the two samples | Methods, Discovery forward MR; Data sources IVW primary estimator and supplementary weighted median and MR-Egger; original GWAS covariate adjustments are source-level, not re-estimated. |
|  | d) | Explain how missing data were addressed | Methods, Instrument selection; Discovery forward MR; Results, Analytic coverage Missing variant fields and unmatched or ambiguous variants excluded; 563 of 564 traits analyzable. |
|  | e) | If applicable, indicate how multiple testing was addressed | Methods, Discovery forward MR; Statistical reporting and software BH/FDR across 564 prespecified discovery comparisons; second AF dataset FDR across six selective tests. |
| 7 | **Assessment of assumptions** | Describe any methods or prior knowledge used to assess the assumptions or justify their validity | Methods, Instrument selection; Discovery forward MR; Results, Six forward associations F statistics, Q, MR-Egger intercept, MR-PRESSO, and cis/trans pQTL interpretation. |
| 8 | **Sensitivity analyses and additional analyses** | Describe any sensitivity analyses or additional analyses performed (e.g. comparison of effect estimates from different approaches, independent replication, bias analytic techniques, validation of instruments, simulations) | Methods, Additional robustness analyses; Statistical reporting and software Strict-threshold, MR-RAPS, leave-one-out, reverse MR, Steiger, cis/trans and colocalization. |
| 9 | **Software and pre-registration** |  | Methods, Statistical reporting and software Software and preregistration are documented in the two subitems below. |
|  | a) | Name statistical software and package(s), including version and settings used | Methods, Statistical reporting and software Python 3.10, R 4.4.1, PLINK 1.9, MRPRESSO 1.0 and mr.raps 0.2; MR-PRESSO simulation count, seed and threshold. |
|  | b) | State whether the study protocol and details were pre-registered (as well as when and where) | Methods, Statistical reporting and software The study protocol was not preregistered. |
|  | **RESULTS** |  |  |
| 10 | **Descriptive data** |  | Results, Instrument reconstruction and analytic coverage Descriptive data and the two-sample considerations are mapped in the subitems below. |
|  | a) | Report the numbers of individuals at each stage of included studies and reasons for exclusion. Consider use of a flow diagram | Methods, Data sources; Instrument selection; Results, Analytic coverage; Figure 1 Original AF sample counts, 473 microbial traits, 91 proteins, instrument flow and one non-analyzable trait. |
|  | b) | Report summary statistics for phenotypic exposure(s), outcome(s), and other relevant variables (e.g. means, SDs, proportions) | Methods, Data sources; original GWAS reports [4, 5, 23, 25] Phenotypic means/SDs are not in the summary-data analysis; refer to the cited source reports. |
|  | c) | If the data sources include meta-analyses of previous studies, provide the assessments of heterogeneity across these studies | Methods, Data sources; original GWAS reports [4, 5, 23, 25] Source-study meta-analysis heterogeneity was not re-assessed from available summary data. |
|  | d) | For two-sample MR:  i.  Provide justification of the similarity of the genetic variant-exposure associations between the exposure and outcome samples  ii.  Provide information on the number of individuals who overlap between the exposure and outcome studies | Methods, Data sources; Discussion, Limitations European-ancestry source datasets are described; exact participant overlap and cross-sample association similarity could not be quantified from available metadata. |
| 11 | **Main results** |  | Results, Six forward associations; Tables 1–2; Additional file 1 Main effect estimates and uncertainty are given in the main and supplementary results. |
|  | a) | Report the associations between genetic variant and exposure, and between genetic variant and outcome, preferably on an interpretable scale | Methods, Instrument selection; Additional file 1, S1–S6 Exposure instrument associations, strength, harmonization and outcome associations are available in supplementary results. |
|  | b) | Report MR estimates of the relationship between exposure and outcome, and the measures of uncertainty from the MR analysis, on an interpretable scale, such as odds ratio or relative risk per SD difference | Abstract, Results; Results, Six forward associations; Tables 1–2 Odds ratios and 95% confidence intervals are given for discovery and selective second-dataset analyses. |
|  | c) | If relevant, consider translating estimates of relative risk into absolute risk for a meaningful time period | Not applicable Absolute AF risk translation was not undertaken from summary-level genetic estimates. |
|  | d) | Consider plots to visualize results (e.g. forest plot, scatterplot of associations between genetic variants and outcome versus between genetic variants and exposure) | Figures 2–3; Figure legends Forest and robustness plots visualize MR estimates and genetic architecture. |
| 12 | **Assessment of assumptions** |  | Results, Six forward associations; Results, Protein cis/trans architecture Assumption checks and their limitations are reported in the subitems below. |
|  | a) | Report the assessment of the validity of the assumptions | Results, Six forward associations; Discussion, Limitations Steiger, MR-PRESSO and MR-Egger findings are reported with explicit caveats. |
|  | b) | Report any additional statistics (e.g., assessments of heterogeneity across genetic variants, such as *I^2^*, Q statistic or E-value) | Results, Six forward associations; Additional file 1, S7 and S14 Cochran Q and MR-PRESSO global and outlier tests are reported. |
| 13 | **Sensitivity analyses and additional analyses** |  | Results, Sensitivity to instrument threshold and MR-RAPS; Figure 3 Strict-threshold, MR-RAPS and related checks are mapped in the subitems below. |
|  | a) | Report any sensitivity analyses to assess the robustness of the main results to violations of the assumptions | Results, Six forward associations; Sensitivity to instrument threshold and MR-RAPS Robustness varies across MR-RAPS and strict-threshold models; limitations are stated. |
|  | b) | Report results from other sensitivity analyses or additional analyses | Results, Second AF GWAS; Protein cis/trans architecture; Additional file 1 Selective second-dataset estimates, protein stratification and FGF-5 colocalization. |
|  | c) | Report any assessment of direction of causal relationship (e.g., bidirectional MR) | Results, Reverse MR; Six forward associations Full reverse screen and approximate observed-scale Steiger directionality findings. |
|  | d) | When relevant, report and compare with estimates from non-MR analyses | Introduction; Discussion Observational AF, microbiome and IL-6 literature are discussed, but no quantitative non-MR comparison was performed. |
|  | e) | Consider additional plots to visualize results (e.g., leave-one-out analyses) | Figure 3; Additional file 1, S7 Leave-one-out and other sensitivity displays. |
|  | **DISCUSSION** |  |  |
| 14 | **Key results** | Summarize key results with reference to study objectives | Discussion, opening paragraph; Conclusions Six conventional IVW signals and uneven sensitivity support are summarized. |
| 15 | **Limitations** | Discuss limitations of the study, taking into account the validity of the IV assumptions, other sources of potential bias, and imprecision. Discuss both direction and magnitude of any potential bias and any efforts to address them | Discussion, Limitations Weak instruments, pleiotropy, overlap, ancestry, selective validation and colocalization limits. |
| 16 | **Interpretation** |  | Discussion Cautious interpretation, mechanism and clinical relevance are mapped below. |
|  | a) | Meaning: Give a cautious overall interpretation of results in the context of their limitations and in comparison with other studies | Discussion, opening and closing paragraphs; Conclusions Hypothesis-generating interpretation and differences across estimators and AF datasets. |
|  | b) | Mechanism: Discuss underlying biological mechanisms that could drive a potential causal relationship between the investigated exposure and the outcome, and whether the gene-environment equivalence assumption is reasonable. Use causal language carefully, clarifying that IV estimates may provide causal effects only under certain assumptions | Discussion, microbial and protein paragraphs Ecological signatures, tissue-specific IL-6 signaling and limits of gene-environment equivalence. |
|  | c) | Clinical relevance: Discuss whether the results have clinical or public policy relevance, and to what extent they inform effect sizes of possible interventions | Discussion, Implications for arrhythmia research; Conclusions No clinical intervention target or established causal pathway is claimed. |
| 17 | **Generalizability** | Discuss the generalizability of the study results (a) to other populations, (b) across other exposure periods/timings, and (c) across other levels of exposure | Abstract, Conclusions; Discussion, Limitations Predominantly European ancestry; timing and exposure-level generalizability remain uncertain. |
|  | **OTHER INFORMATION** |  |  |
| 18 | **Funding** | Describe sources of funding and the role of funders in the present study and, if applicable, sources of funding for the databases and original study or studies on which the present study is based | Declarations, Funding First Hospital of Lanzhou University, grant ldyyyn2020-51; funder had no role in design, analysis, writing or submission decision. |
| 19 | **Data and data sharing** | Provide the data used to perform all analyses or report where and how the data can be accessed, and reference these sources in the article. Provide the statistical code needed to reproduce the results in the article, or report whether the code is publicly accessible and if so, where | Declarations, Data availability statement GWAS Catalog and original source citations; scripts and reconstructed outputs at Zenodo DOI 10.5281/zenodo.22308382. |
| 20 | **Conflicts of Interest** | All authors should declare all potential conflicts of interest | Declarations, Disclosure / conflict of interest statement The authors declare no competing interests. |

Official source: https://www.strobe-mr.org/download/strobe-mr-checklist-fillable-doc/ (STROBE-MR Initiative; checklist CC BY 3.0).
